# A comparative study to evaluate the feasibility of preoperative percutaneous catheter drainage for the treatment of lumbar spinal tuberculosis with psoas abscess

**DOI:** 10.1186/s13018-018-0993-9

**Published:** 2018-11-19

**Authors:** Zhen Lai, Shiyuan Shi, Jun Fei, Guihe Han, Shengping Hu

**Affiliations:** Department of Orthopedics, Hospital of Integrated Traditional Chinese and Western medicine in Zhejiang Province, 208 Huancheng E.Rd, Hangzhou, 310003 Zhejiang Province People’s Republic of China

**Keywords:** Lumbar vertebra, Tuberculosis, Spine, Psoas abscess, Catheter drainage

## Abstract

**Background:**

Spinal tuberculosis is a frequent cause of psoas abscess (PA), and PA largely negates the efficacy of antituberculosis therapy. This study aimed to investigate the clinical outcome of preoperative percutaneous catheter drainage (PCD) in patients with lumbar spinal tuberculosis and PA.

**Methods:**

Between January 2015 and January 2017, 72 patients with lumbar spinal tuberculosis with PA were assigned to group A (preoperative PCD) and group B (*n* = 36 per group). All patients received posterior pedicle screw fixation and anterior focal debridement and fusion. Data on intraoperative blood loss, the duration of the surgery, and the length of the anterior incision were recorded, as well as the postoperative anal exhaust time, visual analogue scale (VAS), Cobb angle, lumbar vertebra function, erythrocyte sedimentation rate (ESR), C-reactive protein (CRP) level, and sinus tract formation.

**Results:**

Sixty-eight patients were followed up for an average time of 13 months (range 6–21 months). Until the final follow-up, no mixed infections, recurrence of tuberculosis, pedicle screw loosening, or screw pullout had occurred. There were significant between-group differences in blood loss, surgery duration, anterior incisional length, postoperative anal exhaust time, and sinus tract formation. As compared with group B, the ESR and CRP levels of the patients in group A were markedly improved following 3 weeks of antituberculosis therapy and 1 week postsurgery.

**Conclusion:**

Preoperative PCD helps to increase the efficacy of antituberculosis therapy prior to surgery, reduce surgical trauma, and avoid postoperative complications, making it a safe and feasible treatment option for lumbar spinal tuberculosis with PA.

## Introduction

Psoas abscess (PA) is a result of myositis, which is caused by many types of bacteria, fungi, parasites, and viruses [1]. PA affects adjacent tissues and has serious public health implications. Spinal tuberculosis is considered a frequent cause of PA in developed countries [2]. According to a recent study, approximately 75–83% of patients with spinal tuberculosis may suffer from a paraspinal abscess or PA [3]. The latter arises when spinal tuberculosis advances through the periosteum, thereby causing inflammation and abscesses.

Chemotherapy plays a significant role in treating spinal tuberculosis. There is evidence that some patients with tuberculous PA may respond to medical therapy, such as antituberculosis treatment, with antituberculosis drugs reaching inhibitory concentrations in lesions [4]. However, a large number of patients fail to respond to medical therapy, necessitating surgical treatment. In recent years, studies have reported good clinical outcomes with posterior pedicle screw fixation combined with anterior focal debridement and fusion in treating lumbar spinal tuberculosis with PA [5, 6]. For instance, after debridement for spinal tuberculosis with a bilateral paraspinal abscess or PA, Li et al. treated the tuberculous lesions with gelatin sponges containing 5 g of streptomycin. They reported that streptomycin was released gradually and that local concentrations of the drug were maintained in the lesions [7]. However, following necrosis, with liquefaction of the PA region and thickening of pus walls, preoperative antituberculosis drugs have difficulty in penetrating the wall of a pyogenic abscess, which often leads to an unsatisfactory outcome. Therefore, in cases of PA, it is best to remove the abscess before surgery to improve the outcome of preoperative antituberculosis therapy.

During the past 10 years, accumulating evidence has strongly suggested that percutaneous catheter drainage (PCD) prior to surgery may be a new practical approach in the management of lumbar spinal tuberculosis with PA [8–10]. As documented previously, PCD is easy to perform, results in less post-surgical pain, and avoids complications associated with anterior surgery. Ye et al. reported that in comparison with anterior PCD, posterior PCD minimized the duration of surgery, reduced surgical trauma, and facilitated the recovery of patients with tuberculous PA [11]. The risk of complications with posterior PCD was also lower than with anterior PCD in these patients. The clinical outcome of preoperative PCD in treating lumbar spinal tuberculosis with PA has been seldom paid attention. The present prospective controlled study investigated short-term outcomes of preoperative PCD in the treatment of lumbar spinal tuberculosis with PA.

## Materials and methods

### Diagnostic criteria for lumbar spine tuberculosis

Lumbar spinal tuberculosis was diagnosed based on a comprehensive medical history, clinical manifestations, imageological diagnosis, as well as staining of pus and microscopy. All the enrolled patients had a history of lumbago, restricted lumbar vertebral function, partial pressing pain, and percussion pain. X-rays and computed tomography (CT) revealed osteoclasia and sequestra, and magnetic resonance imaging (MRI) showed an abscess near the pathological vertebrae and PA. Tuberculosis was confirmed by a staining and microscopic examination of pus drained from the abscess, followed by rapid liquid culture using the BACTEC MGIT 960 system (BD Biosciences, USA) and GeneXpert assay (MTB/RIF, BD, USA).

### Inclusion and exclusion criteria

The inclusion criteria were (1) patients who fulfilled the diagnostic standard for lumbar spinal tuberculosis with PA with an abscess diameter ≥ 3 cm, (2) patients who had indications for spinal tuberculosis surgery (i.e., the presence of spinal compression, a sequestrum, overt bony destruction, and vertebral instability), (3) patients who underwent posterior pedicle screw fixation, anterior debridement, and bone graft fusion, and (4) patients who cooperated with the clinical research and provided signed informed consent.

Patients with one or more of the following were excluded: (1) patients with surgical contraindications, including those with other organ diseases who could not tolerate a long surgery and anesthesia and those with active pulmonary tuberculosis; (2) patients whose surgery had to be postponed because their ESR and CRP levels did not decrease after receiving antituberculosis treatment for 3 weeks; (3) patients with mental illness who could not cooperate with the surgical treatment; (4) patients whose antituberculosis schedules had to be changed during the 3 weeks of treatment; (5) patients who received anterior debridement and bone graft fusion in more than one segment; and (6) patients with a follow-up period less than 6 months.

### General data

In this prospective study, 72 consecutive patients with lumbar spine tuberculosis companied with PA who presented to the Hospital of Integrated Traditional Chinese and Western Medicine in Zhejiang Province (Hangzhou, China) from January 2015 to January 2017 were included. Informed consent was obtained from all the patients and this study was approved by the ethics committee of the Hospital of Integrated Traditional Chinese and Western Medicine in Zhejiang Province. All the patients received a standard antituberculosis regimen containing rifampicin (0.45–0.6 g/d), ethambutol (0.75 g/d), isoniazid (0.3 g/d), and pyrazinamide (1.5 g/d).

The participants were randomly divided into two groups: group A (*n* = 36) and group B (*n* = 36). Briefly, the randomization was performed using opaque sequentially numbered envelopes labeled with the name of the treatment group (group A and B). Briefly, the randomization was performed using opaque sequentially numbered envelopes labeled with the name of the treatment group (group A and B). These envelopes were sealed, shuffled, and then numbered in sequential order. For each new patient entering the study, an envelope was opened. Thus, this procedure avoided the chance of having more patients in one group than another. All the patients in group A underwent preoperative PCD immediately after hospital admission. Four patients in group B were excluded in accordance with the exclusion criterion (4) after 3 weeks of antituberculosis treatment. There were 20 males and 16 females in group A (age range 24–73 years; mean age 42.5 ± 10.2 years) and 18 males and 14 females in group B (age range 23–75 years; mean age 42.3 ± 9.8 years). There were no statistically significances in the sex, age, ESR, CRP levels, or involved segments of the patients (Table 1).

**Table 1:** Comparison of baseline data in the two groups

Groups	Cases (*n*)	Sex		Age (years)	ESR (mm/h)	CRP (mg/L)	The involved segments single biarticulate triarticular		
		Male	Female						
A	36	20	16	42.5 ± 10.2	74.5 ± 8.2	83.4 ± 7.0	23	10	3
B	32	18	14	42.3 ± 9.8	73.3 ± 9.8	82.8 ± 6.1	21	9	2
Statistic		*χ*^2^ = 0.002		*t* = 0.928	*t* = 0.126	*t = 0.268*	*χ*^2^ = 0.328		
*P* value		0.964		0.360	0.901	0.790	0.848		

*ESR* erythrocyte sedimentation rate, *CRP* C-reactive protein

### Operative techniques

**Fig. 1:**
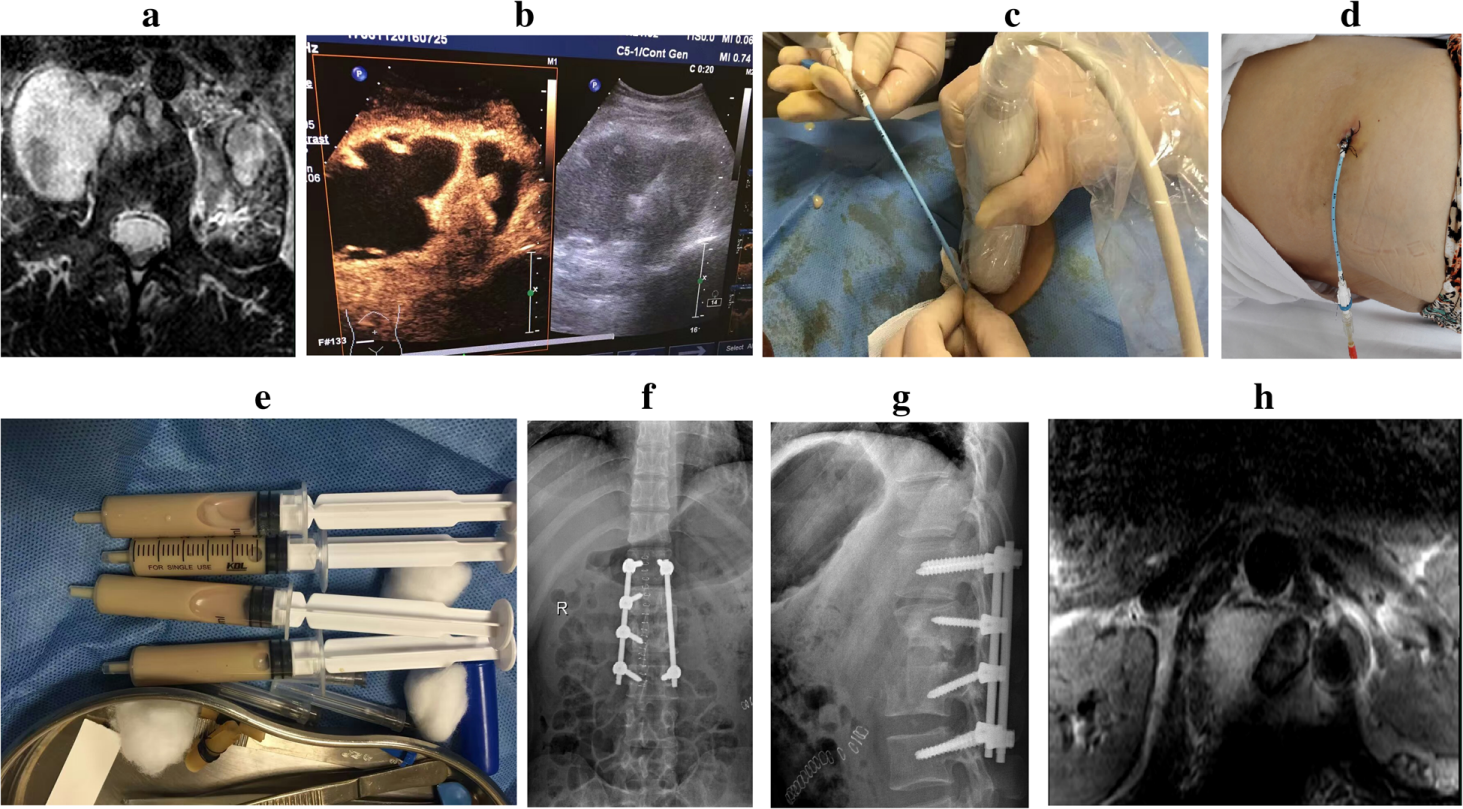
A 46-year-old female patient with tuberculosis of the L1/2 lumbar vertebrae and PA underwent preoperative PCD under ultrasound guidance, antituberculosis therapy for 3 weeks, posterior pedicle screw fixation, and anterior debridement and bone graft fusion. **a**. Preoperative magnetic resonance imaging (MRI) showing lumbar spinal tuberculosis, with abscess formation. **b**–**e**. PCD was performed under ultrasound guidance. **f**, **g**. The X-ray image 3 days after surgery showed good internal fixation and bone grafting. **h**. Postoperative MRI performed at the 3-month follow-up showed complete debridement and no obvious abscess

All patients in group A underwent ultrasound-guided PCD before surgery. Briefly, the location of the maximum diameter of the PA cavity was confirmed by ultrasound guidance after standard skin disinfection procedures and local anesthesia. Puncture needles were then inserted into the abscess cavities. The needle cores were removed when the ultrasound examination confirmed the correct position of the puncture needle. Drainage catheters were inserted into deep abscess to extract pus, followed by a smear test under a microscope to detect acid-fast bacillus. The specimens were detected using the Mycobacterial Growth Indicator Tube 960 (MGIT 960, BD Biosciences, USA) rapid liquid culture method and GeneXpert MTB/RIF assay (Cepheid Inc., USA). Finally, drainage tubes were fixed and connected with drainage bags (Fig. 1). Patients with bilateral PA also underwent bilateral puncture catheter drainage.

**Fig. 2:**
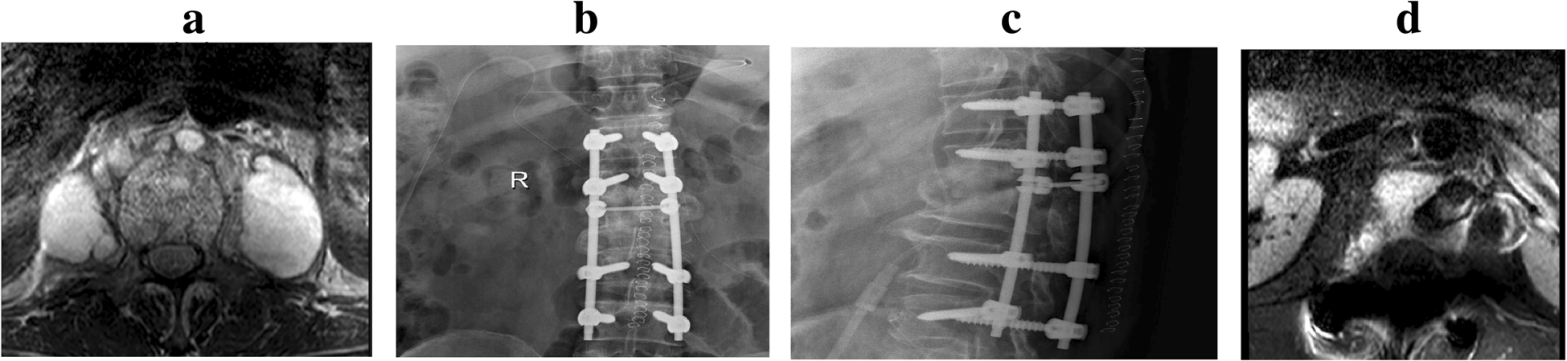
A 39-year-old male patient with tuberculosis of the L1/2 lumbar vertebrae and PA underwent posterior pedicle screw fixation and anterior debridement, with bone graft fusion. **a**. Preoperative MRI showed lumbar tuberculosis, with abscess formation. **b**, **c**. The X-ray image 3 days after surgery showed good internal fixation and bone grafting. **d**. Postoperative MRI at the 3-month follow-up showed complete debridement and no obvious abscess

All patients in both groups were treated with antituberculosis drugs for 3 weeks, followed by posterior pedicle screw fixation and anterior debridement and fusion, which were performed by the same physicians (Figs. 1 and 2). The drainage bags were replaced each day during the 3-week treatment period. In all patients, anterior fusion was achieved using an autogenous bone graft from the iliac crest. Under general anesthesia, a straight incision was made in the middle of the spinous processes, with the patient in a prone position. After pedicle screw fixation, the length of the posterior incision was determined by the fixed segment. The pedicle screws were inserted into the cephalic and caudal adjacent lumbar spinal vertebral segments. The patients were then moved to a lateral position, and an anterior incision was made according to the site of segmental lesions and the size of PA, followed by blunt dissection of the obliquus externus abdominis, obliquus internus abdominis, transverses abdominis along the direction of the fibers. After exposing the peritoneum, the posterior peritoneum and the intestinal canal were pushed away to expose the psoas, reproductive nerves, and ureter. After exposure of the pathological vertebrae using a longitudinal dissociation of the psoas, a pathological examination was performed. Anterior debridement and fusion were conducted, and streptomycin (2 g) and isoniazid (0.6 g) were then administered.

### Observation indexes

The intraoperative blood loss, duration of the surgery, and anterior incision length, as well as the postoperative anal exhaust time, visual analogue scale (VAS) (1 month postsurgery), Cobb angle (1 month postsurgery), ESR and CRP levels (1 week, 1 month and 6 months postsurgery), sinus tract formation and spinal cord injury were evaluated.

The amount of anterior blood loss was considered the blood loss mediated by vacuum aspiration and the blood volume absorbed by the gauze packing. Blood mediated by vacuum aspiration was calculated using the following formula: the amount of liquid in aspirator − (the flushing fluid volume + the amount of pus). The blood volume absorbed by the gauze packing was calculated as follows: the amount of absorbed blood in the gauze packing = the total weight of wet gauze − (the weight of a single gauze pad × the total number of gauze pads).

The duration of surgery was determined as the time of starting cutting the skin until suture of the incision.

Due to the use of preoperative PCD in group A, the severity of PA may have differed in group A and group B. Thus, the length of the anterior incision may have been different. After suturing the anterior incision, the anterior incision length was measured.

The pain index was analyzed using the VAS to evaluate the clinical status of the patients before surgery and one month following surgery. Pains scores of 0, 1–3, 4–6, and 7–10 were judged as no pain, mild pain, moderate pain, and severe pain, respectively.

Before and after surgery, the vertebral segment kyphosis Cobb angle was assessed by X-ray images, with the patient in a standing position. An extension cord was drawn along the upper end plate of the centrum prior to the symptomatic vertebrae, and another extension cord was drawn along the lower end plate of the centrum next to the symptomatic vertebrae. The angle between the two lines was defined as the Cobb angle.

The Frankel grade was used to evaluate the status of spinal cord injury before surgery and at the final follow-up. Grade A denoted complete motor and sensory function disorder; grade B denoted complete function and incomplete sensory function; grade C denoted motor function disorder and incomplete sensory function; grade D denoted useful motor function, with or without auxiliary tools; and grade E denoted no motor or sensory function disorder, with a pathological reflex.

The lumbar vertebral function of the patients was estimated using the Japanese Orthopaedic Association (JOA) score.

### Postoperative management

All patients were postoperatively treated with antituberculosis and liver protection drugs. A compound glycyrrhizin injection was used as the liver protection drug, which was administered via an intravenous injection (20 ml) once a day. Patients with severe anemia and hypoalbuminemia received an erythrocyte suspension and albumin. Pyrazinamide was discontinued 3 months after the operation. After bed rest and immobilization for 12 weeks, all the patients could gradually walk under a protective load.

### Postoperative follow-up and complications evaluation

Sixty-eight patients had a mean follow-up time of 13 months (range 6~ 21 months). The condition of sinus tract formation in group A and group B was analyzed. Six months after surgery, the status of the sequestrum and abscess were assessed through CT examination. Other complications such as mixed infection, recurrence of tuberculosis, pedicle screw loosing, and screw pulling out were evaluated.

### Statistical analysis

Statistical analyses of all data were performed using SPSS, version 16.0 (IBM, Chicago, IL, USA). The measurement data were expressed as the mean ± standard deviation (SD) and compared using Student’s *t* test. A chi-square test was performed to compare the enumeration data. A value of *P* < 0.05 was taken as statistically significant.

## Results

Of 72 cases, four patients with mental illness in group B were excluded due to noncompliance. The other 68 patients had a mean follow-up time of 13 months (range 6–21 months). The average blood loss (*P* = 0.005), duration of the surgery (*P* = 0.003), anterior incision length (*P* = 0.00), and postoperative anal exhaust time (*P* = 0.017) in group A were remarkably lower than those in group B (Table 2). In addition, sinus tract formation was noted in one case in the group and in five cases in group B (Table 2, *P* = 0.002).

**Table 2:** Comparison of anterior blood loss, duration of surgery, anterior incision length, postoperative anal exhaust time, and sinus tract formation in the two groups ($\overline{x}$±s)

Groups	Cases (*n*)	Intraoperative blood loss (ml)	Duration of surgery (minutes)	Incision length (cm)	Anal exhaust time (hours)	Sinus tract formation (*n*)
A	36	156.3 ± 24.7	67.6 ± 13.2	11.7 + 2.6	22.3 ± 5.12	1
B	32	206.5 ± 39.2	105.7 ± 16.3	20.3 + 2.9	30.3 ± 5.69	5
Statistic		*t* = 8.875	*t* = 3.280	*t* = 9.058	*t* = 5.013	*χ*^2^ = 7.696
*P* value		0.005	0.003	0	0.017	0.002

The ESR and CRP of patients in group A were clearly increased 1 week postsurgery but dramatically decreased 1 month and 6 months postsurgery (Table 3, all *P* < 0.05). Variations in the ESR and CRP trends at different follow-up times in group B were in accordance with those observed in group A (Table 3, all *P* < 0.05). As shown in Table 3, the ESR and CRP levels in group A were remarkably lower than those in group B after 3 weeks of antituberculosis therapy and 1 month after surgery. However, there was no statistically significant between-group difference in these parameters 1 month and 6 months postsurgery (Table 3, all *P* < 0.05).

**Table 3:** Comparison of the ESR and CRP level in different follow-up times postsurgery in the two groups ($\overline{x}$±s)

	Group A	Group B	*t* value	*P* value
Cases (*n*)	36	32		
ESR (mm/h)				
3 weeks after anti-TB treatment	37.1 ± 3.2	43.5 ± 5.0	3.012	0.005
1 week after surgery	78.7 ± 0.6	79.3 ± 0.5	3.180	0.003
1 month after surgery	29.1 ± 0.4	28.5 ± 0.7	1.210	0.235
6 months after surgery	9.9 ± 0.7	10.3 ± 0.6	1.794	0.090
CRP (mg/L)				
3 weeks after anti-TB treatment	70.8 ± 0.6	74.2 ± 0.7	3.107	0.004
1 week after surgery	84.3 ± 8.0	93.8 ± 9.1	3.214	0.003
1 month after surgery	15.6 ± 0.8	16.1 ± 0.6	1.816	0.079
6 months after surgery	3.1 ± 0.5	2.8 ± 0.6	1.571	0.126

*ESR* erythrocyte sedimentation rate, *CRP* C-reactive protein, *anti-TB* antituberculous

The results of the VAS indicated that the pain index of the patients in group A and group B was largely improved 1 month following surgery as compared with the preoperative data (Table 4, *P* < 0.05). However, there was no statistically significant between-group difference in the VAS 1 month postsurgery (Table 4, *P* = 0.087). As compared with the measurements before surgery, the Cobb angle of the patients in the two groups was clearly reduced 1 month after surgery (*P* < 0.05), with no significant between-group difference (Table 4, *P* = 0.63).

**Table 4:** Comparison of the VAS and Cobb angle before and after surgery in the two groups ($\overline{x}$±s)

Groups	Cases (*n*)	VAS scoring		Cobb angle (°)	
		Before surgery	1 month after surgery	Before surgery	1 month after surgery
A	36	8.4 ± 0.8	3.2 ± 0.7	18.5 ± 5.5	11.3 ± 4.7
B	32	8.3 ± 0.7	3.5 ± 0.2	17.9 ± 6.1	10.9 ± 5.1
Statistic		0.016	1.725	1.062	0.283
*P* value		0.984	0.087	0.300	0.630

*VAS* visual analog scale

At the final follow-up, the status of spinal cord injury in the patients was evaluated by the Frankel grade. As compared with the preoperative Frankel grades, the spinal cord injury grades of the patients in group A and group B were notably improved at the final follow-up (*P* < 0.01), with no significant between-group difference (*P* > 0.05, Table 5). These findings revealed that the spinal cord injury of all the patients was migrated at the final follow-up.

**Table 5:** Frankel grading in the two groups with lumbar spinal tuberculosis before and after surgery (cases)

	Before surgery		The final follow-up									
			Group A (grades)					Group B (grades)				
Grades	Group A	Group B	A	B	C	D	E	A	B	C	D	E
Grade A	0	0										
Grade B	0	0										
Grade C	6	4				2	4				2	2
Grade D	4	4					4					4
Grade E	26	24					26					24
Summation	36	32				2	34				2	30

The JOA scores, which indicated the lumbar vertebral function of the patients, are displayed in Table 6. In group A, the JOA scores were markedly elevated 1 month postsurgery and at the final follow-up as compared with the presurgery scores (*P* < 0.05). Likewise, the JOA scores of the patients in group B were much higher 1 month postsurgery and at the final follow-up as compared with the scores prior to surgery (*P* < 0.05). When compared with group B, the JOA scores were significantly higher in group A 1 month postsurgery (*P* = 0.036), whereas there was no statistically significant difference in the scores of the two groups at the final follow-up (*P* = 0.782, Table 6). All the patients had recovered lumbar vertebral function 1 month after surgery.

**Table 6:** Comparison of JOA scores in different periods in the two groups ($\overline{x}$±s)

Groups	Cases (*n*)	JOA scoring		
		Before surgery	1 month after surgery	The final follow-up
A	36	6.37 ± 0.51	19.03 ± 3.57	26.71 ± 3.91
B	32	6.41 ± 0.61	16.82 ± 2.75	27.23 ± 5.23
Statistic		0.06	0.128	0.279
*P* value		0.952	0.036	0.782

*JOA* Japanese Orthopaedic Association (JOA)

Postoperative MRI performed at the 3-month follow-up indicated complete debridement and no obvious abscess near symptomatic vertebrae. The CT examination 6 months post-surgery revealed no sequestra and abscess formation in any of the patients, with excellent bone fusion. Up until the final follow-up, there were no cases with mixed infection, recurrence of tuberculosis, pedicle screw loosening, or screw pull out. Typical cases are shown in Figs. 1 and 2.

## Discussion

Clinically, patients with lumbar spinal tuberculosis combined with PA often require surgical treatment to remove the tuberculous lesion, relieve spinal cord compression, enhance the stability of bone grafting and the reconstructed spine, and promote functional recovery [12, 13]. Recently, accumulating evidence has strongly implied that surgical treatment performed during the exudative phase of lumbar spine tuberculosis significantly increased the incidence of abscess recurrence, unhealed lesions, and sinus tract formation [14]. Hence, preoperative antituberculosis therapy has become a necessity for patients with lumbar spinal tuberculosis with PA [15]. In the present study, all the patients were treated with antituberculosis drugs for 3 weeks prior to surgery. As shown by the findings, after antituberculosis therapy for 3 weeks, the ESRs and CRP levels were higher in group A as compared with those in group B, implying that the efficacy of preoperative antitubercular treatment may be influenced by preoperative PCD.

In most cases, antituberculosis drugs administered prior to surgery fail to induce a therapeutic concentration likely to benefit surgical outcomes [16]. In recent years, emerging evidence has suggested that a satisfactory clinical outcome can be obtained by percutaneous abscess drainage puncture guided by ultrasound [17, 18]. The latter is attributed to abscess drainage eliminating inflammatory cytokines, thereby preventing tuberculosis from eroding the tissues around the abscess and improving the outcomes of preoperative antitubercular treatment [19, 20]. Relative to group B, the patients in group A had shorter operative time, less bleeding, and shorter incision length during anterior spine surgery. The aforementioned may be due to preoperative PCD triggering an outflow of large amounts of pus; therefore, the scope of the operation, the difficulty of the surgery, and the surgical trauma were reduced. In addition, the postoperative anal exhaust time and sinus tract formation rate of patients in group A were lower than those in group B. These findings indicate that the reduction in operative trauma in anterior spinal surgery might lead to reduced postoperative complications and improved clinical efficacy. Furthermore, PCD can be completed with the assistance of only a general sonographer. Thus, the procedure can be performed without the need for highly skilled hospital staff, suggesting that the use of PCD could be extended in the clinical setting.

Sometimes, percutaneous PCD fails to thoroughly drain an abscess due to the existence of s thick fester, calcified and caseous tissue in lesions, and a free sequestrum in a partial abscess, which are leading causes of the recurrence of PA. In such cases, anterior focal debridement should be redone. In the present study, we used drainage tubes with a diameter ≥ 3 cm for PCD because undersized drainage tubes are easily blocked by caseous necrotic tissues and surrounding soft tissues, both of which hamper abscess drainage [21]. During surgery, strict implementation of aseptic operative conditions can avoid cross-infection, as well as blood vessel and organ injury, thereby preventing the spread of tuberculosis.

The current study had some limitations. First, the sample size was relatively small. Second, we did not consider the effects of irrigation and local administration of antitubercular agents on the clinical outcome. Lastly, follow-up time was relatively short. Long-term multicenter studies with large sample sizes are needed to objectively and accurately evaluate the outcomes of preoperative PCD.

## Conclusion

We conclude that preoperative PCD is a safe and feasible option for the treatment of lumbar spinal tuberculosis with PA and that PCD could enhance the effect of antituberculosis treatment administered prior to surgery, reduce surgical trauma, and reduce postoperative complications. This study provides support for the use of preoperative PCD in treating lumbar spinal tuberculosis with PA to enhance clinical outcomes.

## Abbreviations

CRP: C-reactive protein
ESR: Erythrocyte sedimentation rate
PA: Psoas abscess
PCD: Preoperative percutaneous catheter drainage
